# Mechanical Properties Enhancement of Dissimilar AA6061-T6 and AA7075-T651 Friction Stir Welds Coupled with Deep Rolling Process

**DOI:** 10.3390/ma15186275

**Published:** 2022-09-09

**Authors:** Pisit Kaewkham, Wasawat Nakkiew, Adirek Baisukhan

**Affiliations:** 1 Graduate Program in Industrial Engineering, Faculty of Engineering, Chiang Mai University, Chiang Mai 50200, Thailand; 2 Department of Industrial Engineering, Faculty of Engineering, Chiang Mai University, Chiang Mai 50200, Thailand; 3 Advanced Manufacturing and Management Technology Research Center (AM2Tech), Department of Industrial Engineering, Faculty of Engineering, Chiang Mai University, Chiang Mai 50200, Thailand

**Keywords:** friction stir welding, deep rolling, tensile strength, residual stress, fatigue life

## Abstract

The main purpose of this research was to enhance the mechanical properties of friction stir welds (FSW) in the dissimilar aluminum alloys 6061-T6 and 7075-T651. The welded workpiece has tensile residual stress due to the influence of the thermal conductivity of dissimilar materials, resulting in crack initiation and less fatigue strength. The experiment started from the FSW process using the 2^k^ full factorial with the response surface methodology (RSM) and central composite design (CCD) to investigate three factors. The experiment found that the optimal rotation speed and feed rate values were 979 and 65 mm/min, respectively. Then, the post-weld heat treatment process (PWHT) was applied. Following this, the 2^k^ full factorial was used to investigate four factors involved in the deep rolling process (DR). The experiment found that the optimal deep rolling pressure and deep rolling offset values were 300 bar and 0.2 mm, respectively. Moreover, mechanical property testing was performed with a sequence of four design types of workpieces: FSW, FSW-PWHT, FSW-DR, and FSW-PWHT-DR. It was found that the FSW-PWHT-DR workpiece had an increase in tensile strength of up to 26.29% and increase in fatigue life of up to 129.47% when compared with the FSW workpieces, as well as a maximum compressive residual stress of −414 MPa.

## 1. Introduction

Friction stir welding (FSW) is a method of joining aluminum alloys. It is referred to as solid-state welding because it fuses metals at temperatures lower than the melting point. FSW has strong metallurgical characteristics compared to other welding methods. There is no loss of mixed materials [1,2]. Furthermore, the technique also does not emit toxic fumes or any forms of radiation that are hazardous to operators. Currently, friction stir welding can efficiently weld the same type of aluminum alloy, which can thoroughly solve the welding quality issue, such as welds of AA6061-T6 [3], AA2024 [4], AA7075-T651 [5], AA5754 [6], etc. This is beneficial to the automotive and aerospace industries, where there is an increasing demand for AA6061 and AA7075 due to their lightweight properties relative to their strength [5,7].

However, dissimilar aluminum alloy welding is required to control the factors of the welding process accordingly. Changes in the mechanical properties and structure of metals are influenced by the heat conductivity of dissimilar materials. Throughout the weld, an uneven strength mechanism arises [8]. The workpiece has tensile residual stress because of the shrinkage following quick quenching. This represents the beginning and expansion of crack initiation and propagation. As a result, the fatigue strength performance of the workpiece is reduced [9,10]. Therefore, optimization of the welding process factor, post-weld heat treatment processes to relieve tensile residual stress, and mechanical surface treatment to increase fatigue strength are required.

A mechanical surface treatment, such as shot peening, laser shock peening, and deep rolling, is used to prevent fatigue and extend the life of a workpiece because it creates compressive residual stress on the workpiece surface due to plastic deformation. The surface structure better resists the tensile stress from the outside, which can reduce or retard the fatigue life of materials [11,12,13,14]. However, the literature revealed that the shot peening technique produces uneven roughness on the workpiece surface [15]. Laser shock peening techniques, resulting in the residual stress of asymmetrically distributed compression, have been proposed [16]. When the effectiveness of mechanical surface treatment processes is compared, deep rolling creates less surface roughness than the shot peening and laser shock peening techniques [17]. Furthermore, the deep rolling procedure generates a deeper and larger residual stress than other methods [18,19]. As a result, the deep rolling procedure is generally acknowledged and used to treat fatigue issues.

However, the deep rolling process requires precise parameter control, especially for dissimilar AA6061-T6 and AA7075-T651 welded by FSW due to differences in material properties. At present, no research has applied the FSW process followed by post-weld heat treatment and the deep rolling process to improve the mechanical properties of dissimilar AA6061-T6 and AA7075-T651 aluminum welded alloys. As a result, this research combined the friction stir welding process, post-weld heat treatment, and deep rolling process to improve the mechanical surface properties of dissimilar 6061-T6 and 7075-T651 aluminum alloys to increase the workpiece fatigue resistance and prolong the fatigue life.

## 2. Materials and Methods

This research used AA6061-T6 and AA7075-T651 aluminum alloys, and the chemical composition measured by the energy-dispersive X-ray fluorescence (EDXRF) method using JEOL model JSX3400R (all units in % *w*/*w*) is shown in Table 1.

### 2.1. Friction Stir Welding Process

From the literature review [20,21,22], the following factors in FSW are often studied: tool pin profile, welding speed, tool rotational speed, axial load, tool tilt angle, and tool material. This research chose the significant factors that directly affect the weld quality for further investigation: rotation speed, feed rate, and type of stirring tool, through the design of the 2^k^ full factorial experiment with RSM in conjunction with CCD to find out the optimal conditions, which are shown in Table 2. The designated response was the tensile strength (MPa).

**Figure 1:**
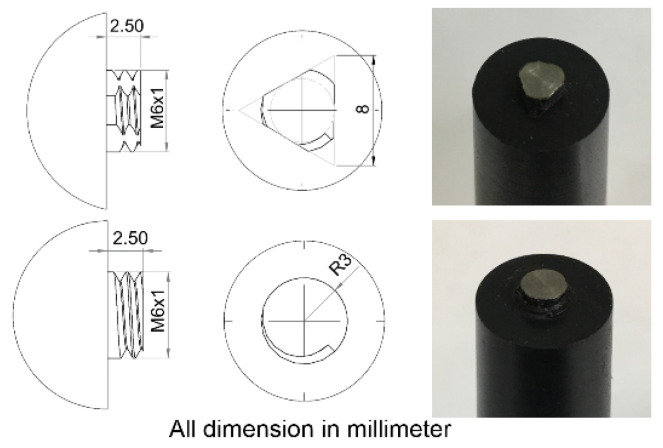
Cylindrically threaded tool with three flat faces (3L) and a cylindrical groove (CG).

**Figure 2:**
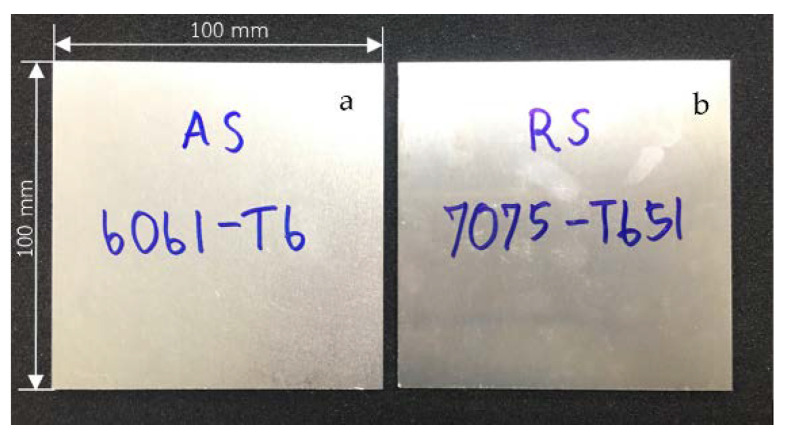
Aluminum plates: (**a**) AA6061-T6; (**b**) AA7075-T651.

**Figure 3:**
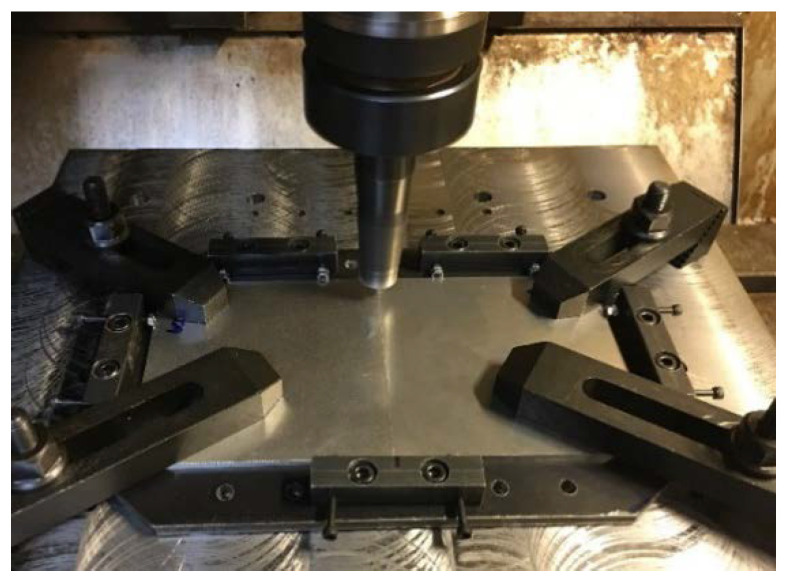
Friction stir welding process.

This research designated the AA6061-T6 aluminum alloy as the advancing side and the AA7075-T651 aluminum alloy as the retreating side [20]. The stirring tool rotated in a counterclockwise direction. The stirring tool was cylindrically threaded with three flat faces (3L) and a cylindrical groove (CG) made of hardened SKD61 steel, as shown in Figure 1. This research used the following aluminum plate dimensions: 3 mm thickness, 100 mm length, and 100 mm width, as shown in Figure 2. This FSW process used the Bridgeport Computer Numerical Control (CNC) machine, model VMC500, as shown in Figure 3.

### 2.2. Post-Weld Heat Treatment Process

Post-weld heat treatment (PWHT) was used to relieve residual stress. In the PWHT process, we used the FSW workpiece with the optimal conditions of the friction stir welding process. From the literature review [23], by studying the mechanical properties of welds of 6061-T6 and 7075-T6 aluminum alloys using a PWHT temperature of 530 °C for 4 h with aging at 140 °C for 6 h, it was discovered that the heat-treated material released residual stress, and the hardness values were evenly distributed throughout the area. Furthermore, in [7,24], the post-weld treatment process could improve the micro hardness of the fusion zone (FZ) and heat affected zone (HAZ) to the level of the original base metal (BM), and the joint strength and ductility. This research used a heat treatment furnace, model TN1000D.

### 2.3. Deep Rolling Process

In the deep rolling process, the FSW-PWHT workpiece was used with the optimal FSW condition. In total, 20 workpieces were designed for this experiment. From the literature review [25,26,27], the following factors in the deep rolling process are often studied: force, ball diameter, number of passes, feed rate, and rolling offset. This research chose the significant factors that are congruent with the research objectives for investigation: pressure, speed, offset, and direction, through the design of the 2^k^ full factorial experiment, which are shown in Table 3. The designated response was the tensile strength (MPa).

**Figure 4:**
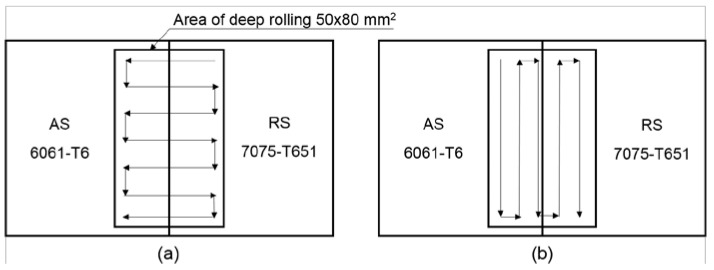
Deep rolling direction: (**a**) transverse direction and (**b**) longitudinal direction.

**Figure 5:**
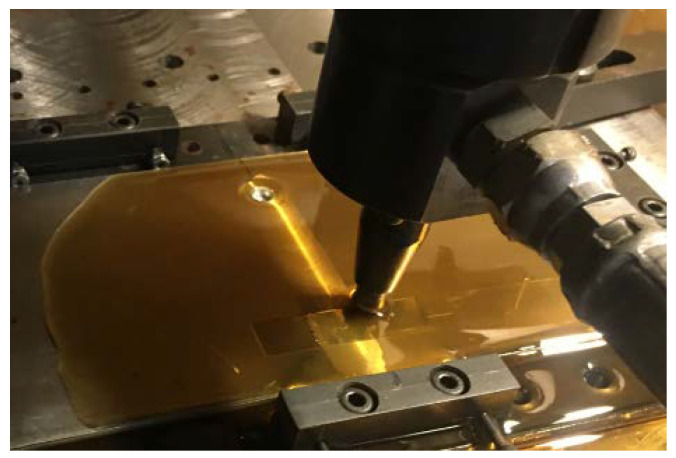
Deep rolling process.

The dimensions of the deep rolling area were 50 mm in width and 80 mm in length, with 2 directions of deep rolling: transverse direction, as shown in Figure 4a, and longitudinal direction, as shown in Figure 4b. An example of the deep rolling process is shown in Figure 5.

### 2.4. Preparing the Workpiece for Testing

**Figure 6:**
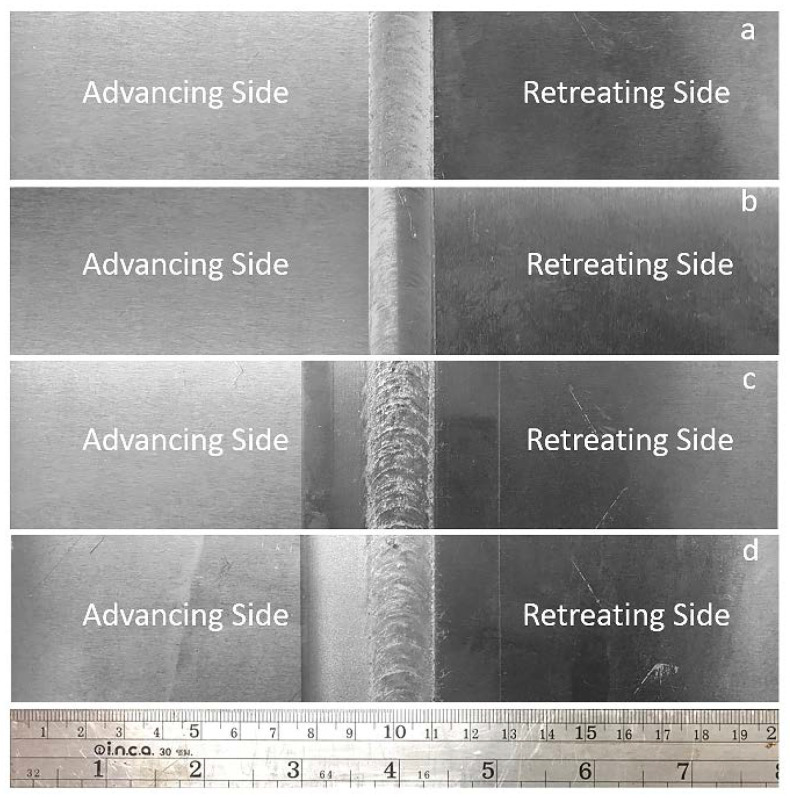
Photographs of the workpieces: (**a**) FSW; (**b**) FSW-PWHT; (**c**) FSW-DR; (**d**) FSW-PWHT-DR.

Photographs of the workpiece are shown in Figure 6. The optimal parameters of the FSW and DR processes were used to create three new workpieces for each design in the sequence, as follows:

1. Only FSW was applied (FSW);
2. FSW was applied, followed by PWHT (FSW-PWHT);
3. FSW was applied, followed by the DR process (FSW-DR);
4. FSW was applied, followed by PWHT, and then the DR process (FSW-PWHT-DR).

### 2.5. Tensile Strength Testing

Tensile strength testing was conducted using a machine from LLOYD Instruments, model LR50K, at a room temperature of about 25 °C, set up with default parameters. The tensile strength workpieces were prepared according to ASTM E8. The main dimensions for this test were a 3 mm thickness, a 57 mm gauge length, and a 19 mm width.

### 2.6. Residual Stress Measurement

**Figure 7:**
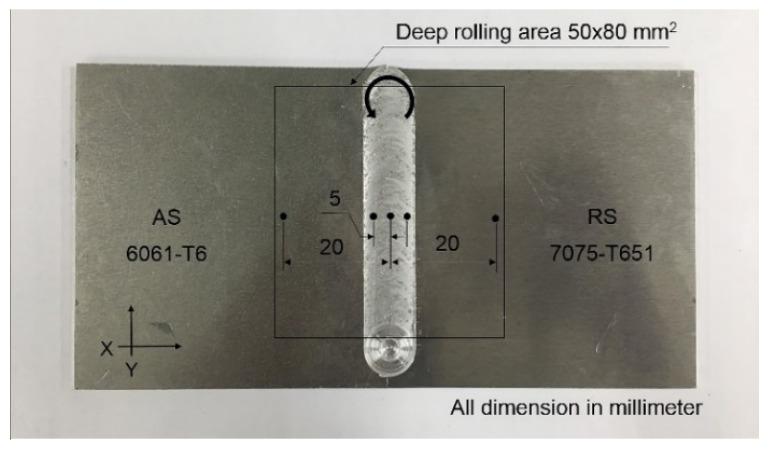
Residual stress measurement position.

Residual stress measurement by X-ray diffraction was conducted using the cos α method. This research used a Pulstec model μ-x360. Residual stress measurement parameters are shown in Table 4. The measurement position of the residual stress was at a distance of 0, ±5, and ±20 mm from the center of the weld, as shown in Figure 7.

### 2.7. Fatigue Testing

**Figure 8:**
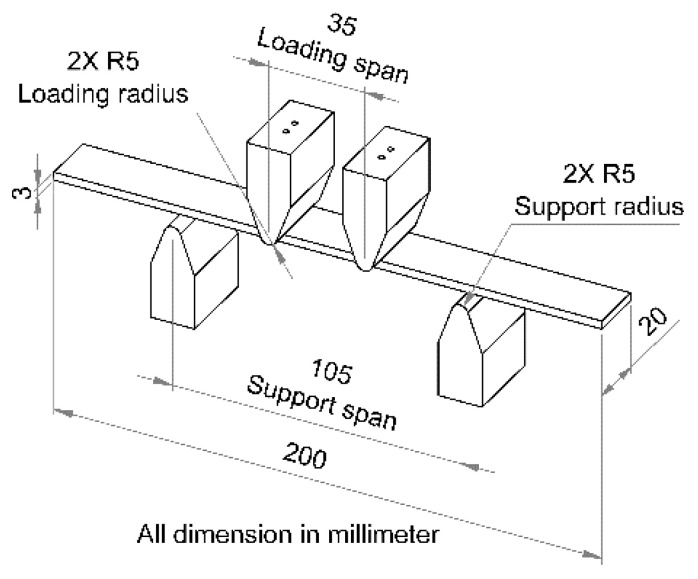
Test workpiece details of ASTM D6272.

According to the ASTM D6272 standard, when undertaking the four-point bending test, the workpiece used a support span of 105 mm, and the loading span was 1/3 of the support span at a distance of 35 mm, as shown in Figure 8, with a bending stress of 410 MPa [28]. The workpiece dimensions were 200 mm in length, 3 mm in thickness, and 20 mm in width, as shown in Equation (1). This research used a test frequency of 3 Hz at a room temperature of 25 °C, with a stress ratio R = 0.

(1) $$\sigma =\frac{FL}{bd^{2}}$$

where

*σ* = bending stress on the outer side (MPa);
*F* = load at defined point on the load deflection curve (N);
*L* = length of support span (mm);
*b* = width of test workpiece (mm);
*d* = thickness of test workpiece (mm).

## 3. Results and Discussion

### 3.1. Friction Stir Welding Results

This research used the 2^k^ full factorial with the response surface methodology (RSM) in conjunction with the central composite design (CCD) method to find out the optimal conditions. The tensile strength testing results of the workpieces are shown in Table 5.

**Figure 9:**
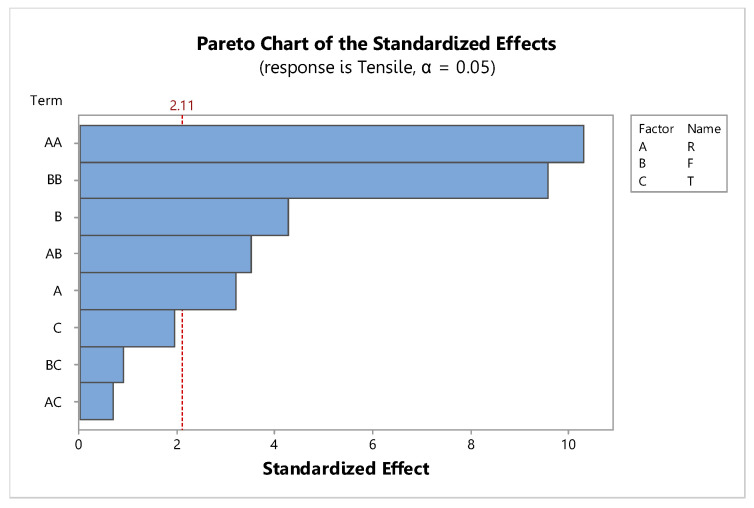
Pareto chart of the standardized effects for friction stir welding.

The factors had significant effects on the tensile strength, with two main effect factors: rotation speed (R) and feed rate (F), with *p*-values of 0.005 and 0.001, respectively, and one interaction effect factor: rotation speed and feed rate (R*F), with a *p*-value of 0.003 and an R-sq of 92.31%, as shown in Figure 9 and Table 6.

This research used a regression equation in uncoded units with an R-sq (pred) of 83.19% to predict the tensile strength of the friction stir welded workpieces, as shown in Equations (2) and (3):

T = −1; Tensile = 218.66 − 1.85R + 2.46F − 8.102R*R − 7.537F*F + 3.65R*F(2)

T = 1; Tensile = 216.42 − 2.85R + 3.80F − 8.102R*R − 7.537F*F + 3.65R*F(3)

where

R = rotation speed (rpm);
F = feed rate (mm/min);
T = type of stirring tool.

**Figure 10:**
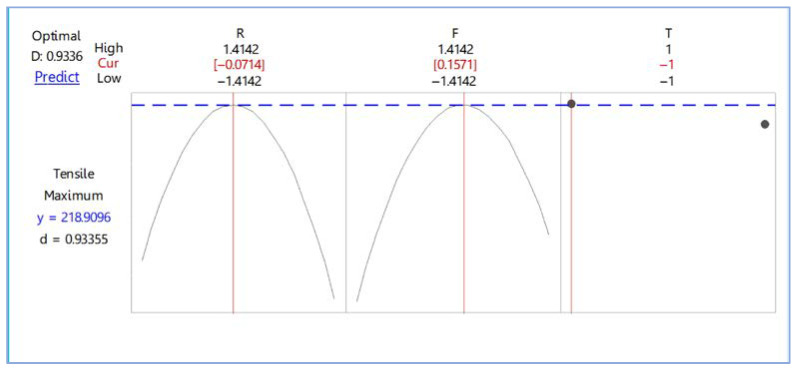
Optimization plot for friction stir welding.

This research used the response optimizer function to find out the optimal condition using the designated maximum tensile strength, as shown in Figure 10. The optimal values were a rotation speed of 979 rev/min and a feed rate of 65 mm/min, with any type of stirring tool. The maximum tensile strength of the workpiece predicted with desirability at 0.815265 was 218.9096 MPa. After that, we confirmed the optimal condition of the five workpieces and found that the tensile strength test average was 218.201 MPa.

### 3.2. Deep Rolling Results

This research used the 2^k^ full factorial to find out the optimal conditions for the deep rolling process. The tensile strength testing results of the workpieces are shown in Table 7.

**Figure 11:**
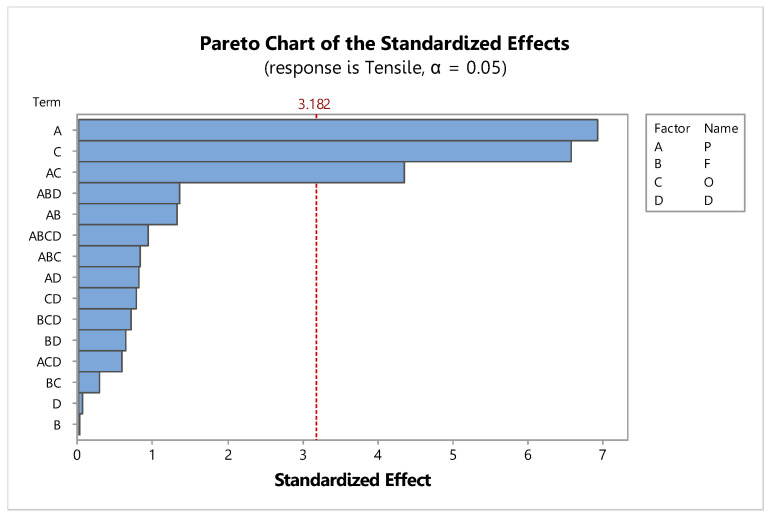
Pareto chart of the standardized effects for deep rolling.

The factors had significant effects on the tensile strength, with two main effect factors—deep rolling pressure (P) and deep rolling offset (O)—with *p*-values of 0.002 and 0.003, respectively, and one interaction effect factor—deep rolling pressure and deep rolling offset (P*O)—with a *p*-value of 0.012 and an R-sq of 96.31%, as shown in Figure 11 and Table 8.

This research used a regression equation in uncoded units with an R-sq (pred) of 82.39% to predict the tensile strength of the deep-rolled workpieces, as shown in Equation (4):

Tensile = 234.70 + 15.66P + 14.86O + 3.01P*F + 9.85P*O − 1.85P*D − 1.43F*D + 1.76O*D + 1.89P*F*O + 3.06P*F*D − 1.33P*O*D − 1.59F*O*D + 2.10P*F*O*D − 5.06Ct Pt(4)

where

P = deep rolling pressure (bar);
F = deep rolling speed (mm/min);
O = deep rolling offset (mm);
D = deep rolling direction.

**Figure 12:**
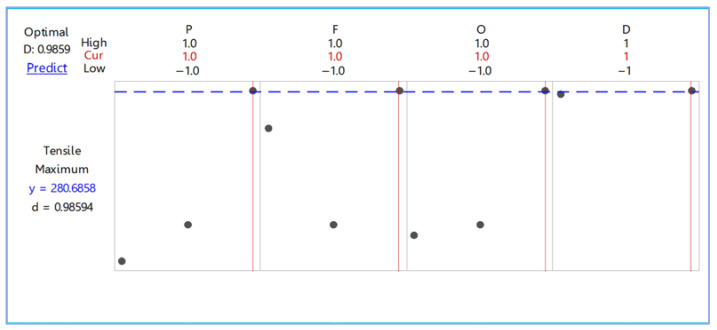
Optimization plot for deep rolling.

This research used the response optimizer function to find out the optimal conditions for the deep rolling process using the designated maximum tensile strength, as shown in Figure 12. The optimal values were a deep rolling pressure of 300 bar and deep rolling speed of 1400 mm/min; any values can be used for the deep rolling offset and deep rolling direction. The maximum tensile strength of the workpiece predicted with desirability at 0.98594 was 280.6858 Mpa. After that, we confirmed the optimal condition of the five workpieces and found that the tensile strength test average was 275.567 MPa.

### 3.3. Tensile Strength Testing Results

**Figure 13:**
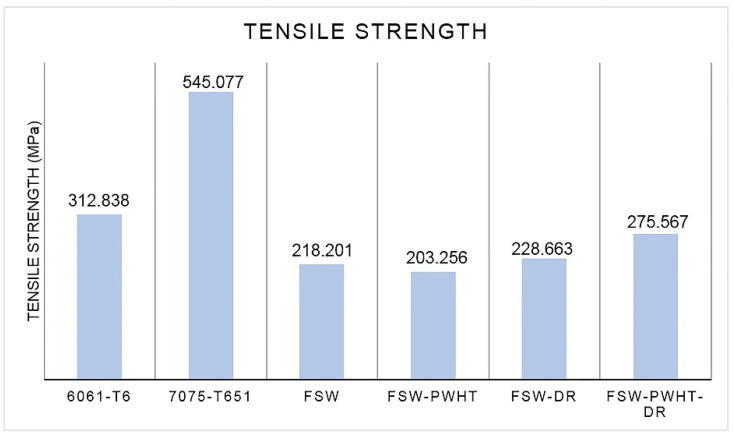
Tensile strength of the workpieces.

**Figure 14:**
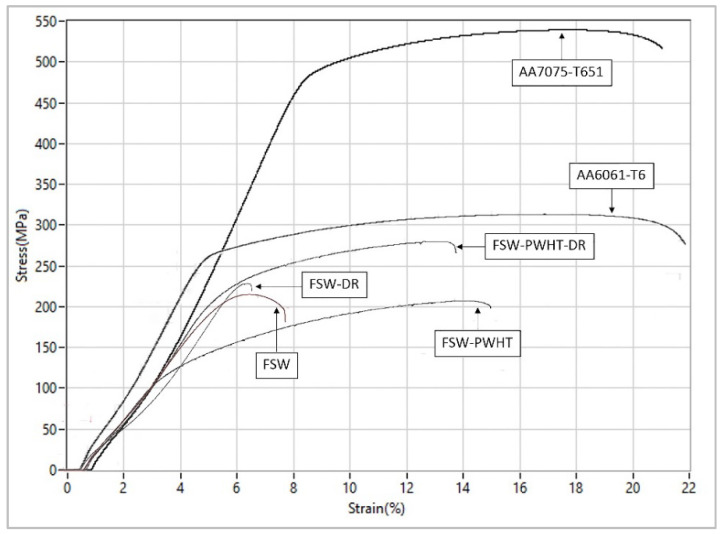
Stress–strain curves of the workpieces.

**Figure 15:**
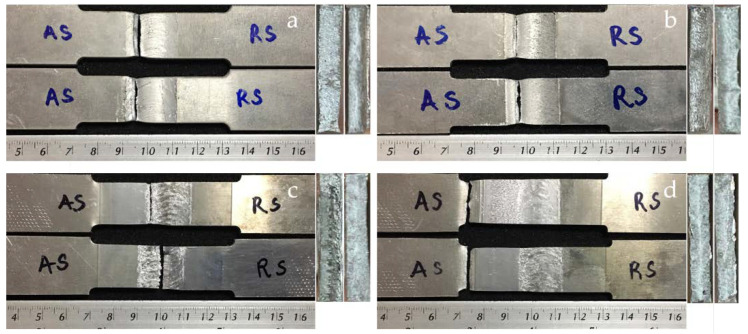
Photographs of the broken workpieces from tensile testing: (**a**) FSW; (**b**) FSW-PWHT; (**c**) FSW-DR; (**d**) FSW-PWHT-DR.

**Figure 16:**
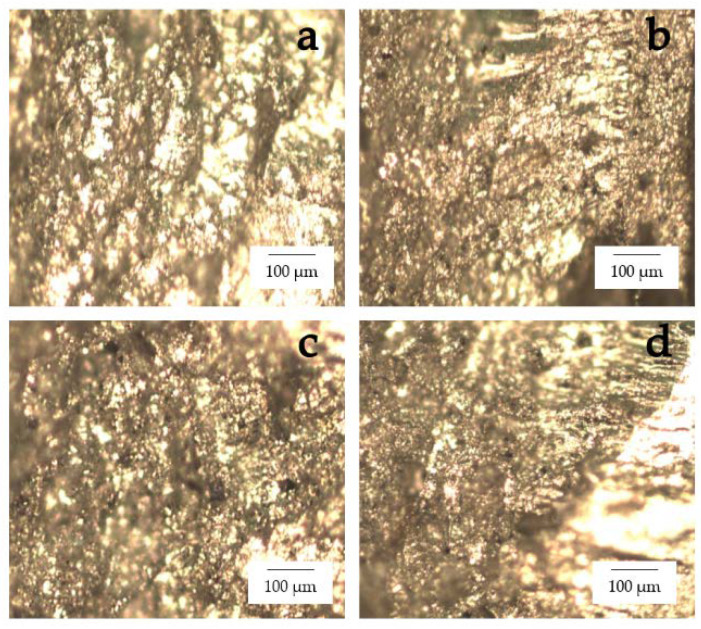
Micrographs of the broken workpieces from tensile testing: (**a**) FSW; (**b**) FSW-PWHT; (**c**) FSW-DR; (**d**) FSW-PWHT-DR.

A sequence of four workpiece designs was used to compare the tensile strengths, as shown in Figure 13. It was found that the tensile strength was 312.838 MPa for the 6061-T6 workpiece, 545.077 MPa for the 7075-T651 workpiece, 218.201 MPa for the FSW workpiece, 203.256 MPa for the FSW-PWHT workpiece, 228.663 MPa for the FSW-DR workpiece, and 275.567 MPa for the FSW-PWHT-DR workpiece, increasing the tensile strength by up to 26.29% when compared to the FSW workpieces that did not undergo any improvement procedures. The stress–strain curves of the workpieces are shown in Figure 14, and photographs and micrographs of the broken workpieces from tensile testing are shown in Figure 15 and Figure 16, respectively. A comparison of the tensile strengths of the workpieces is shown in Table 9. From the literature review [20,29], by investigating the tensile strength of dissimilar materials in friction stir welded joints, it was found that the tool pin profile, feed rate, and tool rotational speed were the most influential factors with significant effects on the tensile strength. The parameterization depends on the mechanical properties of the material. Rotational speeds and feed rates that are too high tend to negatively affect the welding quality, which causes the material to break quickly. On the other hand, heat-treated parts tend to have increased ductility. Following heat treatment, the deep rolling process can be applied. From the literature review [30,31], it was found that the scale of the load and the ball diameter have a significant influence on the tensile strength. In addition, Ref. [32] applied the heat treatment process in combination with the rolling process and found that the AA6056 workpiece had increased ductility and tensile strength. The generation of compressive residual stress in the stir zone (SZ) and thermo-mechanically affected zone (TMAZ) after the PWHT process tends to increase ductility and tensile strength.

### 3.4. Residual Stress Measurement Results

**Figure 17:**
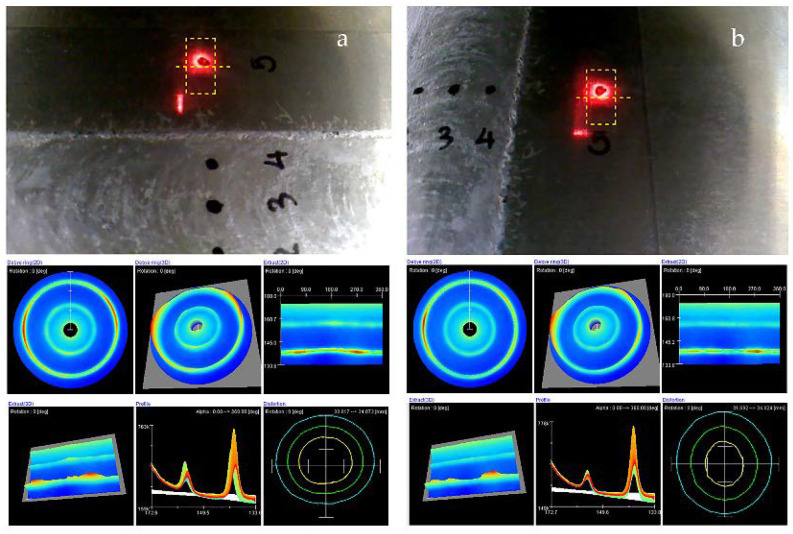
Residual stress measurement for each point: (**a**) transverse direction and (**b**) longitudinal direction.

**Figure 18:**
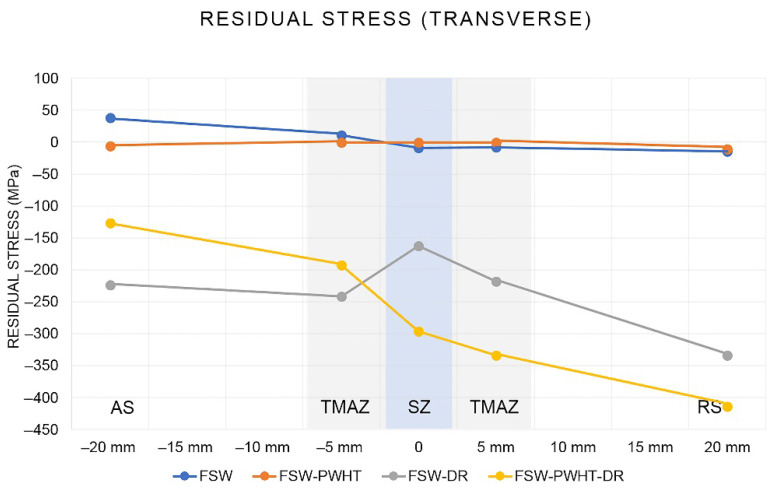
Residual stress in the transverse direction.

**Figure 19:**
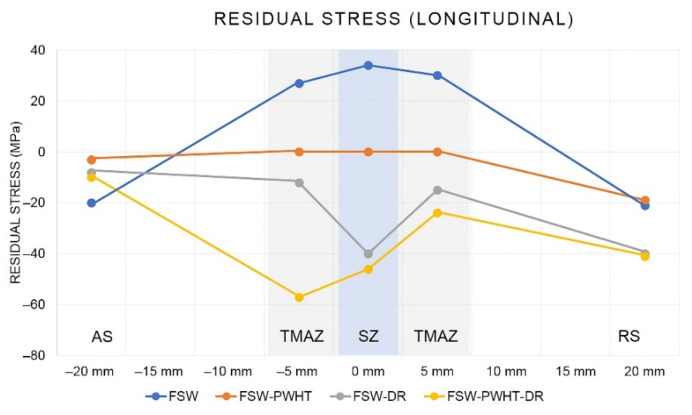
Residual stress in the longitudinal direction.

The residual stress measurement for each point and direction (transverse and longitudinal) is shown in Figure 17. Each measurement point was measured in both the transverse and longitudinal directions. The residual stress measurement results in the transverse direction are shown in Figure 18. It was found that the FSW workpiece had a maximum tensile residual stress (positive value) of +38 MPa at a distance of −20 mm from the center of the weld. When the workpiece was heat-treated and deep-rolled (FSW-PWHT-DR), the workpiece had a maximum compressive residual stress (negative value) of −414 MPa at a distance of +20 mm from the center of the weld, in addition to the remaining compressive residual stress along the entire length of the surface. The residual stress in the longitudinal direction is shown in Figure 19. It was found that the FSW-PWHT-DR workpiece had a maximum compressive residual stress of −57 MPa at a distance of −5 mm from the center of the weld, and the trend of residual stress was the same as in the transverse direction, where the compressive residual stress remained along the entire length of the surface. From the literature review [18,26,33], it was found that the tensile residual stress in the weld area remained after the welding process. This caused the workpiece to initiate a crack. When the workpiece was heated after welding, it was found that the surface residual stress was relieved, and there was a consistent distribution of residual stress throughout the weld. When the workpiece was deep-rolled (FSW-PWHT-DR), it was found that the deep rolling technique resulted in the workpiece having compressive residual stress.

### 3.5. Fatigue Life Results

**Figure 20:**
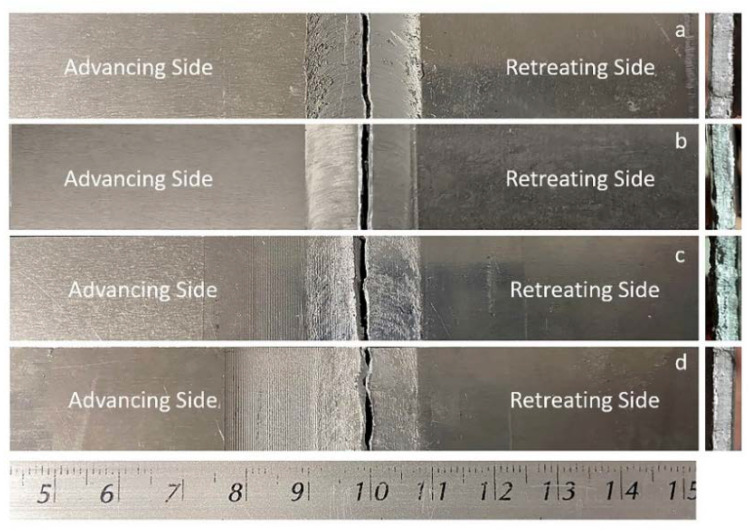
Photographs of the broken workpieces from fatigue life testing: (**a**) FSW; (**b**) FSW-PWHT; (**c**) FSW-DR; (**d**) FSW-PWHT-DR.

**Figure 21:**
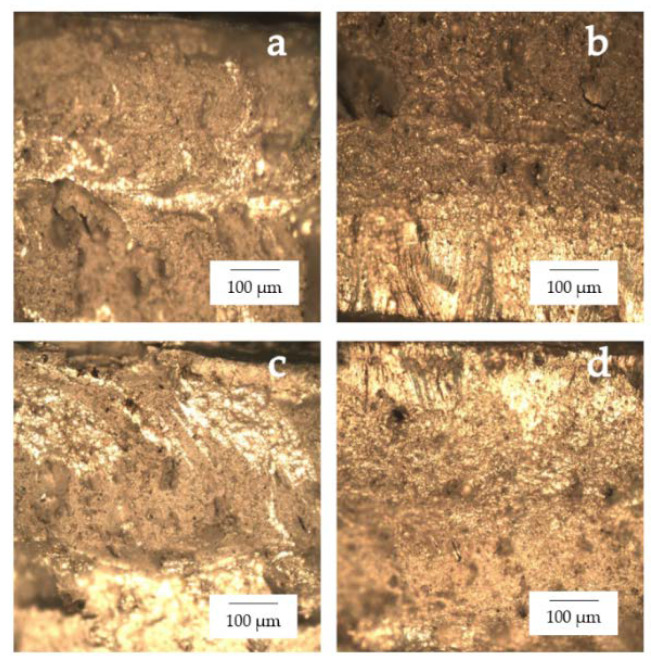
Micrographs of the broken workpieces from fatigue life testing: (**a**) FSW; (**b**) FSW-PWHT; (**c**) FSW-DR; (**d**) FSW-PWHT-DR.

**Figure 22:**
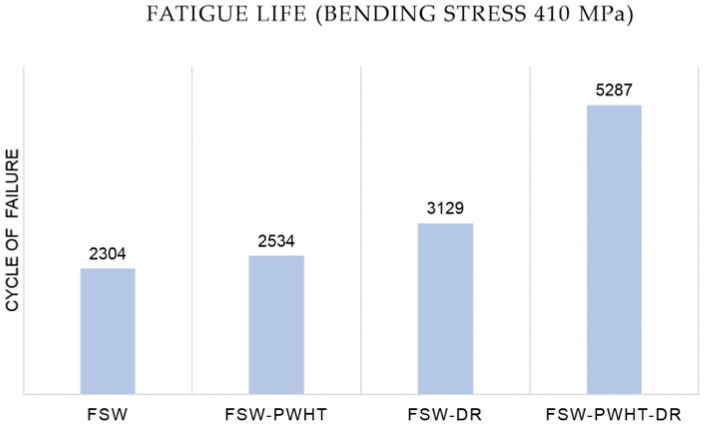
Fatigue life of the workpieces.

Photographs and micrographs of the broken workpieces from fatigue life testing are shown in Figure 20 and Figure 21, respectively. The fatigue life test with a bending stress of 410 MPa is shown in Figure 22. The fatigue life was 2304 cycles for the FSW workpiece, 2534 cycles for the FSW-PWHT workpiece, 3129 cycles for the FSW-DR workpiece, and 5287 cycles for the FSW-PWHT-DR workpiece. The FSW-PWHT-DR workpiece can increase the fatigue life by up to 129.47% when compared with the FSW workpieces that did not undergo any improvement processes. A comparison of the fatigue life of the workpieces is shown in Table 10. The increase in compressive residual stress had a significant influence on the fatigue life. After the FSW process, the welded area had tensile residual stress that tended to result in low fatigue resistance. After the PWHT and DR processes, it was found that the FSW-PWHT-DR workpiece had compressive residual stress that tended to result in high fatigue resistance. In addition, the deep-rolled workpiece’s surface hardness, its surface roughness was uniformly smooth, increasing the fatigue strength [25,34,35]. The compressive residual stress produced by deep rolling tended to result in higher fatigue resistance [18,19].

### 3.6. Microstructure

**Figure 23:**
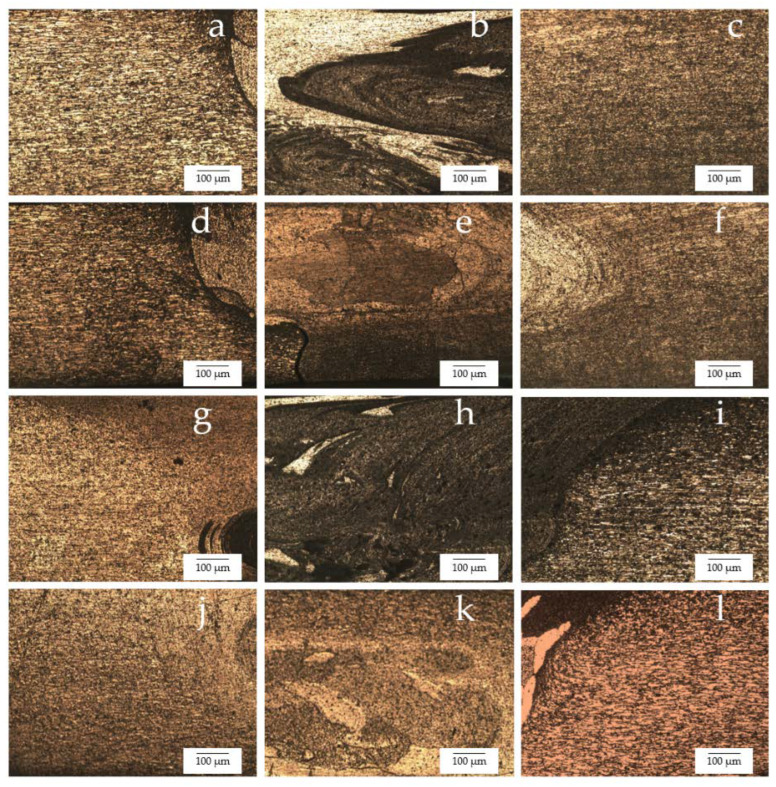
Microstructure of the workpieces: (**a**) TMAZ-AS of FSW; (**b**) SZ of FSW; (**c**) TMAZ-RS of FSW; (**d**) TMAZ-AS of FSW-PWHT; (**e**) SZ of FSW-PWHT; (**f**) TMAZ-RS of FSW-PWHT; (**g**) TMAZ-AS of FSW-DR; (**h**) SZ of FSW-DR; (**i**) TMAZ-RS of FSW-DR; (**j**) TMAZ-AS of FSW-PWHT-DR; (**k**) SZ of FSW-PWHT-DR; (**l**) TMAZ-RS of FSW-PWHT-DR.

**Figure 24:**
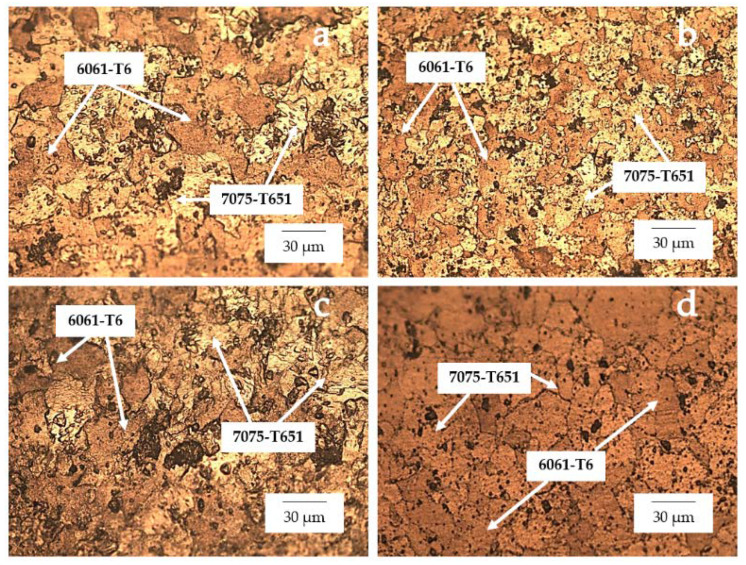
Microstructure of the stir zone (SZ) at 500× (**a**) FSW; (**b**) FSW-PWHT; (**c**) FSW-DR; (**d**) FSW-PWHT-DR.

The optical micrograph of the workpieces is shown in Figure 23. The TMAZ-AS and TMAZ-RS of the FSW, FSW-PWHT, FSW-DR, FSW-PWHT-DR, as shown in Figure 23a,c,d,f,g,i,j,l, respectively, exhibited smaller grain sizes and uniform distributions of precipitates and coarse grain size compared to SZ. The SZ of the FSW, FSW-DR, as shown in Figure 23b,h, respectively, exhibited a layer due to the different types of materials. Furthermore, the SZ of the FSW-PWHT, FSW-PWHT-DR, as shown in Figure 23e,k, respectively exhibited a marginal reduction layer due to the PWHT process. Moreover, the SZ of the FSW, FSW-PWHT, FSW-DR, FSW-PWHT-DR exhibited an equal and fine-grain size for both AA6061-T6 and AA7075-T651, as shown in Figure 24.

## 4. Conclusions

The main purpose of this research was to enhance the mechanical properties of friction stir welds (FSW) in the dissimilar aluminum alloys 6061-T6 and 7075-T651. Therefore, the improvement sequence started from the experiment on the optimal factor for the FSW process. After welding, the workpiece was passed through the PWHT process, and then the DR process experiment was performed on the workpiece to find out the optimal factor. Moreover, the optimal parameters of the FSW and DR processes were used to create a sequence of four design types (FSW, FSW-PWHT, FSW-DR, and FSW-PWHT-DR) for mechanical property testing. The main findings are summarized below:

1. In the friction stir welding process, it was found that the rotation speed and feed rate had significant effects on the tensile strength. The optimal values were a rotation speed of 979 rev/min and a feed rate of 65 mm/min, and any type of stirring tool can be used.
2. In the deep rolling process, it was found that the deep rolling pressure and deep rolling offset had significant effects on the tensile strength. The optimal values were a deep rolling pressure of 300 bar and deep rolling offset of 0.2 mm; the deep rolling speed and deep rolling direction can use any value.
3. The workpiece was subjected to the FSW process, followed by the PWHT process and the DR process (FSW-PWHT-DR). It had a tensile strength of 275.567 MPa and a fatigue life of 5287 cycles, which represent increases of up to 26.29% and up to 129.47%, respectively, when compared with the FSW workpieces that did not undergo any improvement processes. Additionally, the residual stress was converted to compressive residual stress in both the transverse and longitudinal directions.

## Figures and Tables

**Table 1:** The chemical composition of AA6061-T6 and AA7075-T651 aluminum alloys.

Elements	Al	Mg	Si	Fe	Cu	Cr	Zn	Mn	Ti
6061-T6	98.28	1.01	0.63	0.44	0.25	0.11	0.20	0.07	0.02
7075-T651	82.50	3.50	1.39	0.26	2.33	0.30	9.20	0.08	0.00

**Table 2:** Friction stir welding experiments designed using factors, levels, and symbols.

No.	Factor	Unit	Symbol	Level				
				−1.414	−1	0	+1	+1.414
1	Rotation speed	rpm	R	576	700	1000	1300	1424
2	Feed rate	mm/min	F	18	30	60	90	102
3	Type of stirring tool	-	T	-	3L	-	CG	-

**Table 3:** Deep rolling process experiments were designed using factors, levels, and symbols.

No.	Factor	Unit	Symbol	Level		
				−1	0	1
1	Rolling pressure	bar	P	100	200	300
2	Rolling speed	mm/min	F	1000	1200	1400
3	Rolling offset	mm	O	0.1	0.15	0.2
4	Rolling direction	-	D	transverse	-	longitudinal

**Table 4:** Residual stress measurement parameter.

Residual Stress Parameter Items	Process Parameter Values
X-ray tube	Cr-Kα
X-ray detector	Full 2D detector captures the complete Debye ring (visual analysis)
Technique for analysis	Cos α (single position of detector)
Calculation crystal plane	Al (311)
Lattice constant (a)	4.0494 (Å)
Diffraction plane (h, k, l)	311 (FCC)
Collimator size	2 mm
Young’s modulus	263.310 GPa
Poisson ratio	0.34
2θ	139.497°

**Table 5:** Tensile strength test of friction stir welding workpiece.

Std Order	Run Order	Center Pt	Blocks	Factor			Tensile Strength (MPa)
				Rotation Speed (rpm)	Feed Rate (mm/min)	Type of Stirring Tool	
1	1	1	1	−1	−1	−1	204.726
2	2	1	1	1	−1	−1	191.497
3	3	1	1	−1	1	−1	205.573
6	4	1	1	1	−1	1	189.399
9	5	0	1	0	0	−1	220.482
7	6	1	1	−1	1	1	208.857
4	7	1	1	1	1	−1	209.443
8	8	1	1	1	1	1	207.200
10	9	0	1	0	0	1	216.121
5	10	1	1	−1	−1	1	203.186
12	11	0	1	0	0	1	215.764
11	12	0	1	0	0	−1	221.010
17	13	−1	1	0	1.41421	−1	201.211
20	14	−1	1	0	1.41421	1	205.324
22	15	0	1	0	0	1	214.870
19	16	−1	1	0	−1.41421	1	200.437
15	17	−1	1	1.41421	0	−1	200.647
21	18	0	1	0	0	1	214.678
24	19	0	1	0	0	−1	220.646
14	20	−1	1	−1.41421	0	1	201.543
16	21	−1	1	−1.41421	0	−1	204.470
25	22	0	1	0	0	−1	220.426
13	23	−1	1	1.41421	0	1	196.352
18	24	−1	1	0	−1.41421	−1	200.567
26	25	0	1	0	0	1	214.654
23	26	0	1	0	0	−1	216.759

**Table 6:** Analysis of variance for friction stir welding.

Source	DF	Adj SS	Adj MS	F-Value	*p*-Value
R	1	88.13	88.129	10.23	0.005
F	1	156.79	156.794	18.21	0.001
T	1	32.51	32.507	3.78	0.069
R*F	1	106.79	106.792	12.40	0.003
R*T	1	4.02	4.020	0.47	0.504
F*T	1	7.13	7.128	0.83	0.376
**Model Summary**					
**S**	**R-Sq**			**R-Sq (Adj)**	**R-Sq (Pred)**
2.87	92.31%			89.89%	83.19%

**Table 7:** Tensile strength test of the deep-rolled workpiece.

Std Order	Run Order	Center Pt	Blocks	Factor				Tensile Strength (Mpa)
				Rolling Pressure	Rolling Speed	Rolling Offset	Rolling Direction	
4	1	1	1	1	1	−1	−1	226.839
9	2	0	1	0	0	0	−1	240.769
12	3	0	1	0	0	0	1	225.115
10	4	0	1	0	0	0	1	222.135
1	5	1	1	−1	−1	−1	−1	213.334
6	6	1	1	1	−1	1	−1	272.776
8	7	1	1	1	1	1	1	281.649
5	8	1	1	−1	−1	1	1	244.456
2	9	1	1	1	−1	−1	1	222.224
11	10	0	1	0	0	0	−1	230.546
3	11	1	1	−1	1	−1	1	213.140
7	12	1	1	−1	1	1	−1	220.025
18	13	1	1	1	1	−1	1	227.879
20	14	1	1	−1	1	1	1	216.876
13	15	1	1	1	1	1	−1	276.870
19	16	1	1	1	−1	1	1	268.986
15	17	1	1	−1	−1	1	−1	214.867
17	18	1	1	1	−1	−1	−1	225.657
14	19	1	1	−1	−1	−1	1	215.778
16	20	1	1	−1	1	−1	−1	213.879

**Table 8:** Analysis of variance for deep rolling.

Source	DF	Adj SS	Adj MS	F-Value	*p*-Value
P	1	3922.67	3922.67	47.87	0.002
F	1	0.05	0.05	0.00	0.981
O	1	3533.56	3533.56	43.12	0.003
D	1	0.36	0.36	0.00	0.950
P*F	1	144.65	144.65	1.77	0.255
P*O	1	1552.14	1552.14	18.94	0.012
P*D	1	54.57	54.57	0.67	0.460
F*O	1	6.77	6.77	0.08	0.788
F*D	1	32.72	32.72	0.40	0.562
O*D	1	49.41	49.41	0.60	0.481
P*F*O	1	57.19	57.19	0.70	0.450
P*F*D	1	149.84	149.84	1.83	0.248
P*O*D	1	28.50	28.50	0.35	0.587
F*O*D	1	40.51	40.51	0.49	0.521
P*F*O*D	1	70.77	70.77	0.86	0.405
**Model Summary**					
**S**	**R-Sq**			**R-Sq (Adj)**	**R-Sq (Pred)**
6.77	96.31%			91.24%	82.39%

**Table 9:** Comparison of the tensile strengths of the workpieces.

Workpiece	6061-T6	7075-T651	FSW	FSW-PWHT	FSW-DR	FSW-DR-PWHT
Tensile strength	+43.37%	+149.81%	0%	−6.85%	+4.79%	+26.29%

**Table 10:** Comparison of the fatigue life of the workpieces.

Workpiece	FSW	FSW-PWHT	FSW-DR	FSW-PWHT-DR
Fatigue life	0%	+9.98%	+35.81%	+129.47%

## Data Availability

Not applicable.
